# Voice First as a Platform for Addressing and Measuring Social Isolation

**DOI:** 10.1093/geroni/igaa057.2221

**Published:** 2020-12-16

**Authors:** Ryan Elza

**Affiliations:** AARP Foundation, Washington, District of Columbia, United States

## Abstract

Voice First solutions offer the opportunity to promote social connectedness and independence. AARP Foundation has been successful in deploying Voice First devices (Amazon Alexa, Google Home Assistant) in senior living and affordable housing communities and developing Voice First solutions through its Connect2Affect Connected Communities program. Connected Communities empowers older adults through voice first solutions to help them obtain the social support resources needed to stay socially connected, remain independent and age in place. AARP Foundation has developed three Voice First solutions 1) Community Hub, a voice application that encourages participation in group activities by voice-enabling a community’s calendar of events and allowing older adults to discover, register and receive reminders for events, 2) A data collection voice application that administers the 10-item version of the Duke Social Support Index (DSSI) at regular intervals to assess an older adults’ condition of isolation, 3) Social Check-in, a guided risk assessment for social isolation that helps older adult understand their individual risk factors.

